# Teasing, Taunting, and the Politics of Politeness: High Sociometric Status Is Associated with Expectation-Consistent Behavior

**DOI:** 10.1371/journal.pone.0104737

**Published:** 2014-08-26

**Authors:** Michael W. Kraus, Christopher Oveis, Maria Logli Allison, Randall C. Young, John Tauer, Dacher Keltner

**Affiliations:** 1 Department of Psychology, University of Illinois, Urbana-Champaign, Champaign, Illinois, United States of America; 2 Rady School of Management, University of California San Diego, San Diego, California, United States of America; 3 Department of Psychology, University of California, Berkeley, California, United States of America; 4 Department of Psychology, Bridgewater College, Bridgewater, Virginia, United States of America; 5 Department of Psychology, University of St. Thomas, Saint Paul, Minnesota, United States of America; Institut Pluridisciplinaire Hubert Curien, France

## Abstract

Research examining face-to-face status hierarchies suggests that individuals attain respect and admiration by engaging in behavior that influences others' judgments of their value to the group. Building on this research, we expected that high-status individuals would be less likely to engage in behaviors that violate group norms and expectations, relative to low-status individuals. Adolescent participants took part in an interaction in which they teased an opposite-gender friend (Study 1) or an experiment in which taunting or cheering expectations were manipulated (Study 2). Consistent with the hypothesis, high-status boys and girls engaged in teasing behaviors consistent with their gender roles, relative to their low status counterparts (Study 1). In Study 2, high-status boys engaged in more direct provocation and off-record commentary while taunting, and more affiliative behavior while cheering on their partner, relative to low-status boys. Discussion focused on how expectation-consistent actions help individuals maintain elevated status.

## Introduction

Understanding how sociometric status hierarchies influence social behavior is important for social relationships among adults as well as adolescents, and is a vibrant area of research [1]–[3]. Drawing from recent theoretical advances suggesting that group status is functionally based—that is, based on group judgments of the individual's capacity to bring coherence and value to the group [1]–[3]—we test the hypothesis that individuals with high status in face-to-face social groups are more likely to engage in behavior consistent with group-based expectations, relative to their low-status counterparts. We argue that engaging in expectation-consistent behavior allows high-status individuals to gain and maintain their elevated social positions. In the present research, we test this hypothesis by examining nonverbal behavior in social interactions that involve teasing (Study 1) and taunting (Study 2), because such behaviors are guided, at least in part, by politeness norms and expectations.

### A Functional Approach to Sociometric Status

Sociometric status is defined as the prestige, prominence, and respect that individuals attain in their face-to-face social groups [4]. Recent theory and research indicate that people who are conferred elevated sociometric status by their peers tend to achieve this social position by demonstrating their value to their social group [1]–[3]. Specifically, whereas dominance-based patterns of behavior (e.g., coercion, aggression) may damage one's reputation, and reduce respect among one's peers [3], behaviors that demonstrate an individual's tendency to have the goals of the group in mind lead to enhanced social status.

Importantly, this theorizing predicts that status is granted to individuals who act in ways that enable the smooth functioning of the group, that maximize the well-being of group members, and more generally, embody social norms and expectations that make for coherent groups. For example, individuals typically rise in status in hunter-gatherer societies according to the extent that they can provide protein rich nutrients through hunting [5]. Individuals also attain high status when they demonstrate competence in group tasks. For example, individuals working in teams who demonstrated more competent behaviors during group conversations (e.g., volunteering answers to the group, making suggestions) attained higher status [6]. High status is also conferred on individuals who show greater investment in the group as a whole [7]. For instance, individuals who contributed more to collective resources during an economic game were seen as having higher status by the other members of their social group [8]. Similarly, work in actual organizations suggests that individuals who develop reputations as helpful co-workers are judged as higher in status by their peers, relative to individuals who help others less [9].

### Expectation-Consistent Actions Maintain Status

High-status individuals maintain their elevated social positions through a number of means. For instance, recent survey research suggests, for example, that high-status individuals justify their social positions through endorsing meritocratic beliefs—beliefs that elevated social positions are based on hard work, talent, and effort [10]–[11].

A functional perspective on social status suggests that high sociometric status individuals will best maintain their social status by indicating their value to their social groups. One way in which high-status individuals accomplish this is through engagement in actions that are consistent with the norms, values, and expectations of members of one's social group. Actions consistent with social group expectations should maintain social status for several reasons: First, conforming promotes group solidarity, and convinces group members that one does not have competing loyalties or affiliations [12]. Second, conforming to group norms or expectations suggests that individuals understand rules for group members' behavior ([13]; for similar claims on the benefits of conforming to group norms, values, and expectations, see [14]–[15]). Third, conformity helps people repair bonds with social groups that have rejected them: For example, following an experience of rejection, men who felt the most rejected were also more likely to ingratiate themselves with the group by conforming to group standards (e.g., expressed intentions to donate money to the group, or to help an authority figure), relative to men low in rejection sensitivity [16].

Indirect evidence provides initial support for our status and expectation-consistent action hypothesis: For example, participants role-playing as supervisors placed in situations with people-centered (versus product-centered) expectations were more likely to perceive the individual characteristics of their low-status co-workers—consistent with the role expectations of the situation [17]. As another example, high-status individuals tended to be more extraverted in team-oriented work environments and more conscientious in individual-oriented technical work environments, consistent with the norms and expectations of their specific work teams [18]. In the current research, we sought empirical evidence in support of our status and expectation-consistent action hypothesis by examining teasing and taunting interactions in face-to-face groups of adolescents.

### Teasing, Taunting, and Politeness Theory

Teasing and taunting interactions offer a compelling test of the status and expectation-consistent behavior hypothesis because they are complex social interactions that shift dramatically according to group-based rules and expectations [19]–[20]. In addition, since teasing and taunting do not explicitly indicate competence or cooperation, high-status individuals' behaviors in these interactions can be attributed, at least partially, to a desire to conform to group expectations, rather than status motives related to appearing competent [1].

Politeness theory [19] offers a systematic account of teasing and taunting, differentiating between two kinds of communication. Direct, on-record communication is relevant, truthful commentary that is to be taken literally as a statement of fact, such as when a medical doctor provides news to a patient. Very often, direct commentary risks harming others' esteem or jeopardizes the communicator's reputation as a considerate individual. In these circumstances, individuals often resort to a second kind of communication: indirect, off-record commentary. Indirect acts of communication violate the rules of direct communication with a variety of politeness tactics (e.g., exaggeration, laughter) suggesting non-literal interpretations of the communication. Thus, off-record commentary renders the communication less hostile.

Within this framework, teasing and taunting can be defined as off-record forms of provocative commentary [20]. Teasing is an off-record provocation that comments on something of relevance to the target, but again is accompanied by playful off-record markers, such as shifts in prosody and pacing or the use of metaphor and exaggeration [20]–[21]. Taunting is a challenge, often physical, that incorporates direct provocation with off-record markers that render the challenge less serious.

In the present investigation, we examined informal commentary in taunting and teasing interactions. In Study 1, opposite-gender pairs of friends engaged in a teasing interaction in which they generated a funny nickname about their friend [21]. In Study 2, pairs of boys at a basketball camp were assigned to experimental conditions in which the group expectations were either to taunt or cheer another boy while engaging in a basketball shooting contest. Across studies, we tested the overarching hypothesis that high-status individuals would engage in taunting and teasing behaviors consistent with the group expectations in that context. Specifically, we expected high-status individuals to taunt and tease others during social interactions consistent with expectations based on gender stereotypes (Study 1) or experimentally defined expectations of interaction (Study 2). In addition to testing our hypothesis, the present research advances prior research by assessing status based on outside observer or peer ratings (rather than self-reports), and by assessing the taunting and teasing behavior of adolescents in naturalistic group interactions.

## Study 1: Teasing between Opposite Gender Friends

Gender roles and expectations govern many differences between men and women in their nonverbal behavior during social interactions [22]–[26]. In general, women in Western cultures, when believing that their behavior is being monitored, tend to engage in behaviors that confirm stereotypes about women. For instance, women showed more stereotypically feminine behaviors when they presumed that a male interviewer or potential date was evaluating them [27]–[28]. Similarly, a meta-analytic review indicated that women tended to smile more than men, fitting with gender expectations about affiliation and expressiveness, and this difference was moderated by how explicitly the rules for gender-appropriate behavior were made salient to participants [25]. The above research indicates that gender stereotypic behavior can be thought of as consistent with group-based expectations.

These findings suggest that gender should moderate the relationship between status and the content of teasing. Within mixed-gender interactions (which make gender expectations salient, see [29]), one would expect high status males to tease in a gender-appropriate fashion—using more expressions of directly hostile nonverbal behaviors and actions [22], [26], [30]. High status females within those interactions, by contrast, should tease in ways guided by gender roles made salient by the context—they should tease in a more affiliative, playful fashion, relative to their low status counterparts.

### Methods

#### Participants and procedure

Dyads of opposite-gender friends were recruited from a public high school of approximately 2300 students in Sacramento, California. Friendship nomination forms were distributed by teachers to all 9^th^ and 12^th^ graders. Of those, 16 pairs of 9^th^ graders (*M* = 14.77 years, *SD* = 0.55) and 17 pairs of 12^th^ graders (*M* = 17.92 years, *SD* = 0.44) participated in the laboratory teasing interaction described below. This study and consent procedures were approved by the institutional review board of the University of California, Berkeley. Written consent was obtained from parents or legal guardians of all participants under the age of 18.

Participants were instructed to name and rank their opposite-gender friends in order of closeness. From these responses, we identified opposite-gender students who listed each other in one of the top three positions, and asked them to participate in the study, which took place in an empty classroom at the high school. The opposite-gender friends engaged in a teasing interaction in which they were assigned one of two pairs of initials (H.F. or A.D.), generated a nickname for their friend based upon the initials, and told a story that justified the nickname based on either real or fictional events (for full description, see [31]). One 9^th^ grade male never produced a nickname or a story and thus his dyad was not included in the final analyses. As a result, data analyses were conducted on 32 friendship dyads.

#### Determination of status

Based on sociometric studies of status and peer popularity [32]–[33], each participant received a status score based upon the number of times he or she was nominated as someone else's close friend on the nomination form. The average girl's status score was 4.24 (*SD* = 2.65) and the average boy's status score was 4.82 (*SD* = 3.80), *t*(32) = 0.93, *ns*. Sociometric studies suggest that measures of status indexing the construct based on peer ratings of popularity, though conceptually distinct, are empirically related to other measures of status indexing the construct through peer ratings of respect and prominence [3], [32].

#### Behavioral coding

Each participant's behavior in the role of teaser was coded by two coders. Across all teases, the mean length of time for a tease was 26.24 seconds (26.76 seconds for females and 25.72 seconds for males).

Coders recorded each occurrence of a harsh verbal statement about the person (appearance, personality, or behaviors). The frequency of these coded behaviors was summed into an index of the direct and provocative nature of the tease (*M* = 0.63, *SD* = 0.91). Reliability, calculated by examining the intraclass correlation for coder frequencies of direct provocation, was high (ICC = .74).

Coders scored the teasing interaction by rating the playfulness of the teasing story on a 1 (*not at all*) to 5 (*extremely*) scale (*M* = 2.23, *SD* = 0.70). Reliability, calculated by examining the intraclass correlation for coder ratings of playful behavior, was high (ICC = .83).

### Results and Discussion

Because there was relative non-independence in the teasing behavior of participants (*ICC(1) = *.12 to .43) within dyads in the current sample, we used the actor-partner interdependence model (APIM) in our analysis [34]. The APIM is a multilevel model [35], that has several notable features that make it ideal for conducting analyses using participant data nested within dyads. Most importantly, the model tests associations between one predictor variable of an actor with one or more actor outcome variable while simultaneously estimating the association with the same partner predictor variable within the dyad [34]. The APIM is flexible in its capacity to assess associations between variables in dyads that are distinguishable by gender, like in Study 1, or indistinguishable as in Study 2 [34]. As well, like more typical linear regression analyses, the APIM allows for the systematic testing of interaction terms between predictor variables, so long as the lone predictors are also included in the model simultaneously [36]. In this APIM analysis, we predicted direct provocation and playful teasing behavior using gender, actor- and partner-status, and the interaction between actor-status and gender.

**Figure 1:**
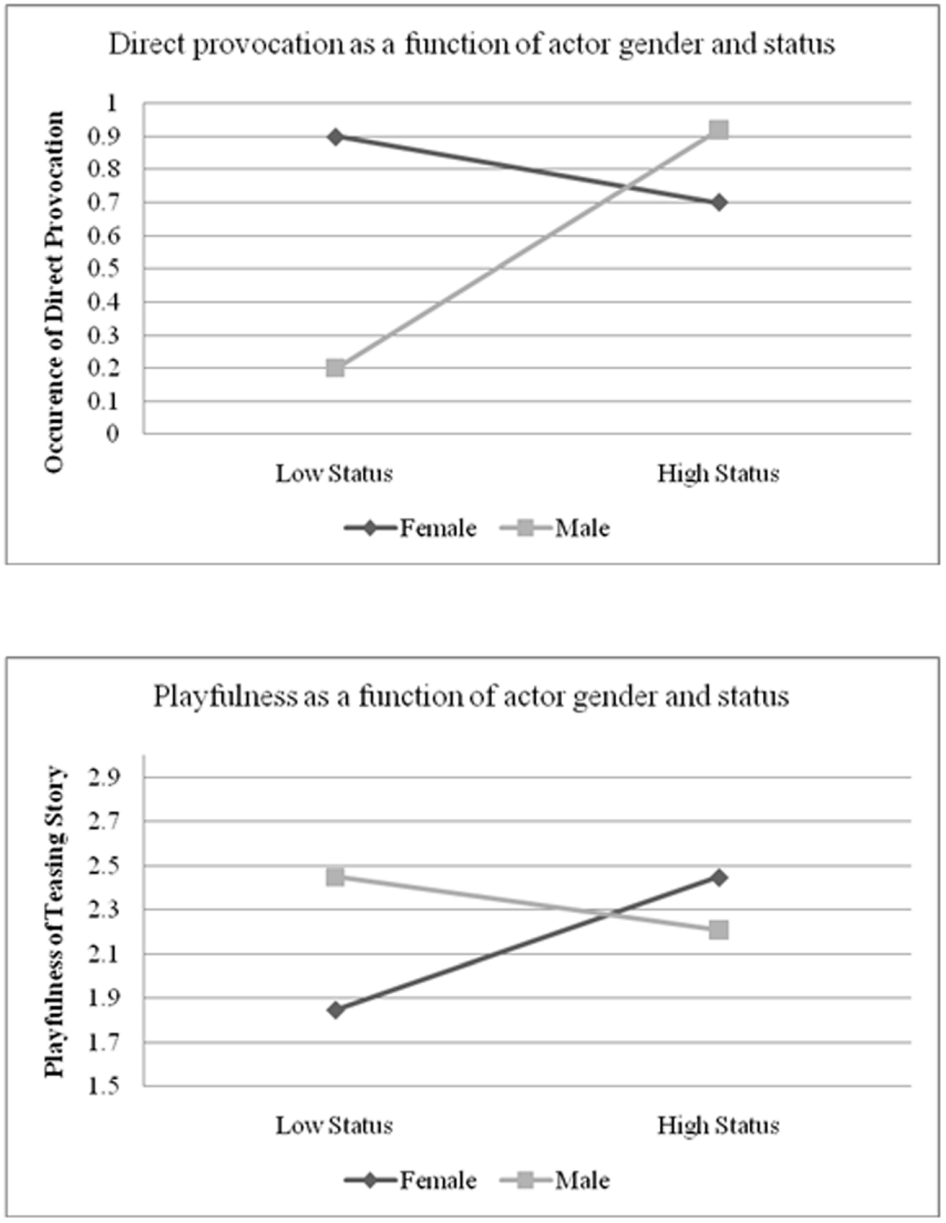
The top panel displays the frequency of direct provocation as a function of gender and participant status (defined as +/− one standard deviation above or below the mean). The bottom panel displays coder ratings of the playfulness of the teasing story as a function of gender and participant status (Study 1).

For our main prediction we expected that, consistent with their gender roles during the interaction, high-status boys would tease using more direct provocation whereas high-status girls would tease using more playful behavior. This is, in fact, what we observed. For direct provocation, a significant interaction emerged between gender and actor-status, *b* = 0.23, *t*(26.73) = 2.53, *p*<.05. A simple slopes analysis revealed that boys with high status teased in more direct, hostile ways, *b* = 0.36, *t*(31) = 2.55, *p*<.05, whereas girls showed no such association between status and direct, hostile teasing, *b* = −0.10, *t*(31) = −0.71, *ns.* This pattern is reflected in the top panel of Figure 1, where high and low status are indexed as one standard deviation above and below the mean respectively. No other effects were significant. For playful teasing behavior, as expected, a significant interaction emerged between gender and actor-status *b* = −.21, *t*(46.77) = −2.23, *p*<.05. A simple slopes analysis revealed that high-status girls tended to tease more playfully, *b* = 0.30, *t*(31) = 2.12, *p*<.05, whereas status was unrelated to playful teasing behavior for boys, *b* = −0.12, *t*(31) = −0.85, *ns.* The bottom panel of Figure 1 shows this pattern, with high and low status indexed as one standard deviation above and below the mean respectively. No other effects were significant.

Consistent with our hypothesis, gender moderated the relationship between status and direct provocation during teasing interactions. More specifically, whereas higher-status boys engaged in directly hostile forms of teasing, higher-status girls teased in a more playful fashion. These findings align with other research examining gender differences in status-based nonverbal behaviors, suggesting that high status among men is associated with gender-appropriate expressions of directly hostile nonverbal behaviors and actions [22], [26], [30], whereas the high status of women is associated with engaging in more gender-appropriate behaviors that include being more likeable [37] and more affiliative and playful [38].

## Study 2: Manipulation of Taunting and Cheering Expectations at a Basketball Camp

In Study 2, we sought to build on the initial evidence by experimentally manipulating behavioral social norms and expectations of boys at a week-long basketball camp. We assigned boys to one of two contexts with differing explicit behavioral social expectations: one that required taunting, and a second that required cheering for another camper. We predicted that relative to low-status boys, high-status boys would be more likely to engage in expectation-consistent behavior, taunting with more direct provocation and off-record behavior in the taunting context—consistent with expectations for teasing and taunting behavior [20], and cheering with more playful behavior in the cheering context.

### Methods

#### Participants and procedure

Eighty volunteers participated in one of two week-long summer basketball camps. The first week of camp consisted of 60 boys in 7^th^ through 9^th^ grade (12–14 years of age), 26 of whom participated in the study. The second week of the camp consisted of 70 boys in 4^th^ through 6^th^ grade (8–11 years of age), 54 of whom participated. This study and consent procedures were approved by the institutional review board of the University of California, Berkeley. Written consent was obtained from parents or legal guardians of all participants under the age of 18.

Campers were paired with another boy of the same age whom they had never met. The taunting or cheering took place at one station called “the pressure cooker” during two consecutive days at the camp. Participants took turns in attempting to make a 15-foot shot. Participants performed this task in pairs, with each member taking a single shot, one day shooting prior to the partner, and on the other day shooting after the partner. The shooter's partner had to remain in a 2′×2′ taped square placed to the left of the shooter. Half of the pairs were randomly assigned to the taunt condition, the other half to the cheer condition. Pairs remained in the same condition on both days of the pressure cooker.

To manipulate taunting expectations, each participant in the taunt condition was told that his partner was a fan rooting against the shooter, and that the fan should try his best to distract the shooter. In the cheer condition, participants were told that the partner was a fan rooting for the shooter, and that the fan should try to encourage the shooter.

#### Behavioral coding

Two coders analyzed each participant's behavior as a fan, beginning immediately after the experimenter handed the ball to the shooter until the shot was attempted.

Coders recorded each occurrence of the following direct forms of provocative behavior: insincere verbal encouragement, harsh verbal statements about the person/performance, harsh vocalizations, hostile/intimidating gestures/physical movements, arm/hand waving in a manner that gets in the way of the shooter, direct stares, hostile facial display, and tongue protrusions. These behaviors were summed into a measure of direct taunting (*M* = 6.04, *SD* = 6.30). Reliability, calculated by examining the intraclass correlation for coder frequencies of direct provocation, was high (ICC = .79).

Following previous studies (e.g., [21], [39]), the frequencies of the following off-record behaviors were coded: deviations from normal pitch, repetition of words/phrases, metaphor, and storytelling/scenario framing. These behaviors were summed into an index of off-record markers (*M* = 2.43, *SD* = 2.78). Reliability, calculated by examining the intraclass correlation for coder frequencies of off-record markers, was high (ICC = .60).

The frequency of the following affiliative behaviors was coded: verbal encouragement, sincere clapping, dancing behavior, arm/hand waving in a friendly manner, and sincere non-verbal celebration/encouragement. These behaviors were summed into an index of affiliative behavior (*M* = 3.11, *SD* = 3.54). Reliability, calculated by examining the intraclass correlation for coder frequencies of affiliative behavior, was high (ICC = .61).

#### Social status

Each boy's social status was rated by two coaches at the basketball camp on both days. Following other studies of status [32], coaches rated how much “respect, influence, and leadership” (1 = *none at all*, 7 = *very much*) each boy had in his specific subgroup in the camp. This item has been found to have excellent predictive validity in other studies of status, correlating with peer assessments of the target's status [32]. To establish reliability, each boy was also rated on the same scale by the supervisor of the camp. Coach and supervisor ratings correlated significantly on both days, *rs* = .72 to .73, *ps*<.01. The status of boys at the camp was then indexed by the average of the two-day coach ratings (*M* = 4.01, *SD* = 1.57).

### Results and Discussion

As in Study 1, we conducted analyses using the APIM for indistinguishable dyads, to account for non-independence in behavior between dyad members (*ICC(1) r* = .40 to .64, *ps*<.01) [34]. First, we expected that taunting expectations would engender greater direct provocation relative to cheering ones, particularly for high-status boys. For this analysis we predicted direct provocation using our experimental manipulation of expectations (taunting coded as “1” and cheering coded as “−1”) and age (older boys coded as “1” and younger boys coded as “−1”), along with standardized variables for actor- and partner-status, and finally, the two-way interactions between actor-status and condition and partner-status and condition. Here, we found a condition main effect suggesting that in the taunting expectations condition there was more direct provocation than in the cheering condition, *b* = 4.22, *t*(35) = 8.39, *p*<.01. We also found an effect for actor-status, *b* = 1.48, *t*(53.33) = 2.99, *p*<.01, and partner-status, *b* = 1.01, *t*(53.33) = 2.04, *p*<.05, such that higher actor or partner status predicted more direct provocation.

**Figure 2:**
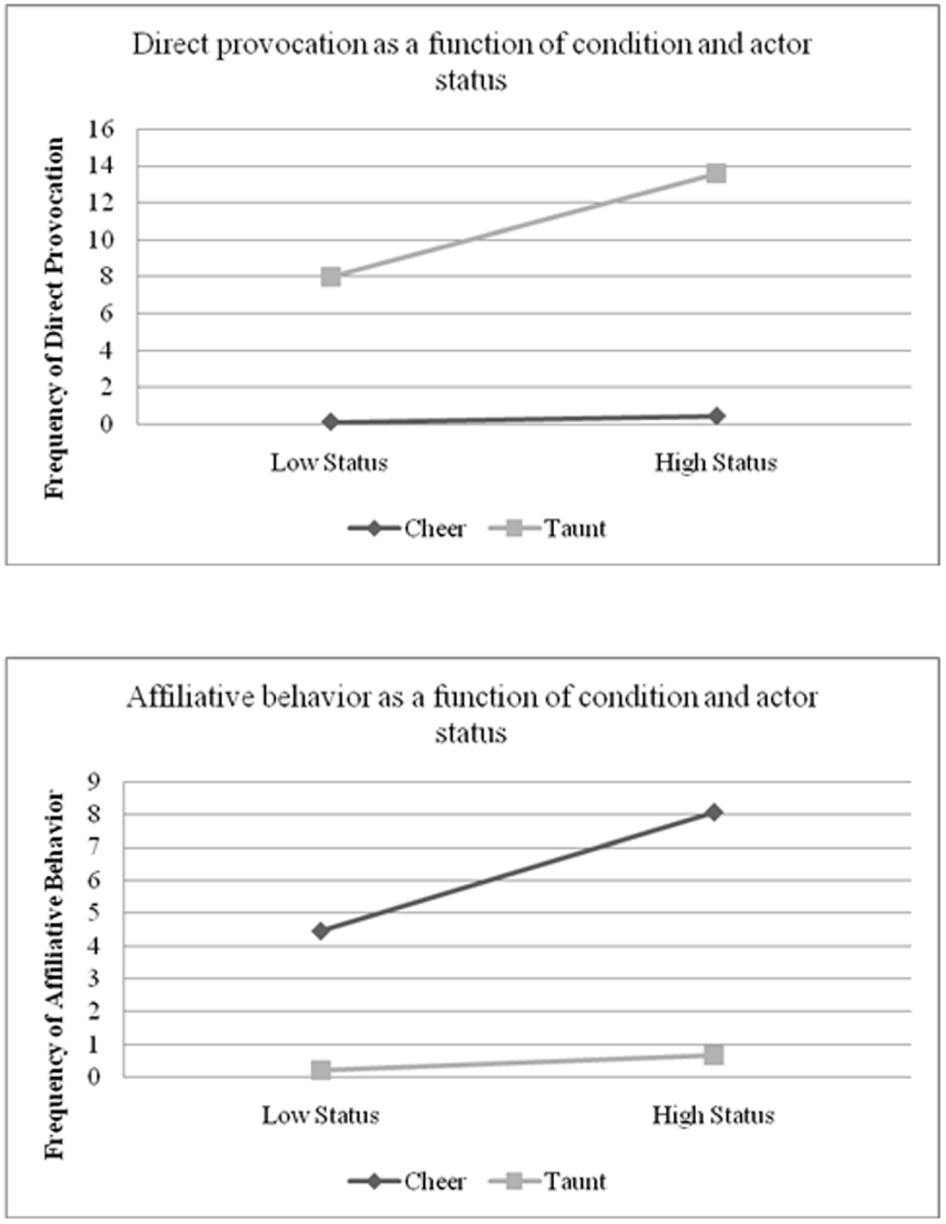
The top panel displays the frequency of direct provocation for participants cheering or taunting the shooter as a function of participant status (defined as +/− one standard deviation above or below the mean). The bottom panel displays the frequency of affiliative behavior for participants cheering or taunting the shooter as a function of participant status (Study 2).

The predicted interaction between condition and actor-status emerged, *b* = 1.32, *t*(52.68) = 2.61, *p*<.05. Examination of simple slopes revealed a pattern in line with our hypothesis: In the taunting condition higher-status participants were more likely to engage in direct provocation relative to their lower-status counterparts, *b* = 2.80, *t*(36) = 4.32, *p*<.01. No such relationship emerged in the cheering condition, *b* = .47, *t*(40) = 0.61, *ns* (see top panel of Figure 2, where status is indexed as plus or minus one standard deviation surrounding the mean). Interestingly, the interaction between condition and partner status was also significant, *b* = 1.04, *t*(52.68) = 2.06, *p*<.05, suggesting that participants engaged in more direct provocation in the taunting condition particularly when their partner was of high status. Age was not significantly associated with direct provocation, *b* = 0.63, *ns*.

Next, we examined whether taunting expectations would yield more off-record forms of commentary, particularly for high-status individuals. In addition to the condition, *b* = 1.18, *t*(35) = 4.15, *p*<.01, and status, *b* = 0.71, *t*(55.89) = 2.49, *p*<.05, main effects, the predicted interaction between condition and actor-status was significant, *b* = 0.63, *t*(55.18) = 2.16, *p*<.05, such that high-status boys were particularly likely to engage in taunting using off-record forms of commentary. No other effects were significant.

Finally, we examined whether high-status boys behaved in more affiliative fashion than low-status boys in the cheering condition. In this analysis, main effects for condition, *b* = −2.58, *t*(35) = −7.14, *p*<.01, actor-status, *b* = 1.02, *t*(46.84) = 2.99, *p*<.01, and partner-status, *b* = 0.75, *t*(46.84) = 2.18, *p*<.05, emerged. However, these effects were qualified by the predicted interaction between condition and actor-status, *b* = −0.79, *t*(46.38) = −2.27, *p*<.05. Aligning with predictions, an analysis of simple slopes revealed that status was significantly positively associated with affiliative behavior in the cheering condition, *b* = 1.81, *t*(40) = 3.42, *p*<.01, such that higher-status boys were particularly likely to engage in affiliative behaviors when cheering, whereas no relationship emerged in the taunting condition, *b* = .23, *t*(36) = 0.51, *ns* (see bottom panel of Figure 2, where status is indexed as plus or minus one standard deviation surrounding the mean). No other effects were significant. That taunting expectations were associated with more direct provocation and off-record commentary among high-status individuals whereas cheering expectations were associated with more affiliative behaviors aligns with our hypothesis that high-status individuals are more likely than their low-status counterparts to engage in behaviors consistent with context-specific norms and expectations.

Interestingly, in an unpredicted finding, participants were more likely to taunt high-status boys using direct provocation relative to low-status boys. We interpret this finding as suggesting one of two things: (1) Boys felt more comfortable directly provoking high-status boys because these boys could handle the taunting due to their elevated status; or (2) Partners of high-status boys were paying those boys back for actual or anticipated direct provocations of their own. As this finding was unexpected, we caution interpreting the results without further examination.

## General Discussion

Functional theories of sociometric status in face-to-face groups suggest that an individual's status is conferred by one's peers, and is based on judgments of one's value to the group [1], [3]. Research indicates that high-status individuals maintain their elevated social positions by cultivating group perceptions of competence [6], or by engaging in actions that suggest strong commitment to the group [9]. In the present study we tested the hypothesis that high-status individuals are also likely to engage in actions consistent with group expectations, because such actions promote group solidarity [12], communicate an understanding of group norms and values [13], and repair group relationships [16]. We tested this hypothesis in the context of teasing and taunting interactions between face-to-face groups of adolescents.

Consistent with our predictions, in Study 1, high-status boys and girls engaged in behavior consistent with their gender roles—that is, high-status boys teased in more directly hostile ways, whereas high-status girls teased in more playful ways. In Study 2 we manipulated group expectations, finding that boys engaged in more spontaneous behavior consistent with these expectations: When instructed to cheer, high-status boys engaged in more affiliative behavior than their low-status peers; when instructed to taunt, high-status boys engaged in more direct provocation and off-record commentary, consistent with politeness theory analyses of teasing [19].

These findings extend what is known about the functional bases of status, suggesting that expectation-consistent actions are associated with positions of elevated status in face-to-face social groups. The current research also extends what is known about status associations with behavior to new samples (adolescents), new kinds of social interactions (teasing and taunting), and a focus on spontaneous verbal and nonverbal behavior. Importantly, the findings were observed using peer and observer ratings of status, suggesting that expectation-consistent behaviors associated with high-status individuals can be attributed to the actual group status of these individuals, rather than to self-report biases [40].

The findings also point out the utility of considering differences in behavior motivated by distinct forms of social hierarchy. Often constructs like social power (e.g., control and freedom), socioeconomic status (e.g., income and education), and sociometric status (e.g., prestige and respect) are conflated in research, obscuring how these unique forms of status influence behavior. In this research, we measured sociometric status in face-to-face groups—wherein a person's social position is conferred by other group members [3]. Status that is conferred by peers is likely to elicit behaviors that show one's value to the group, and as such, group-based motives, or motives to behave consistent with group norms, values, and expectations are likely to be associated with this form of social status. In contrast, the elevated control and freedom arising from social power is likely to engender self-interested action [41]–[42], and resistance to situation-specific influences on behavior [43].

### Limitations and Future Directions

The current findings suggest that high-status individuals are likely to engage in expectation-consistent action. Study 2 is particularly strong in this regard, because situation-specific expectations of spontaneous behavior were experimentally manipulated, and high-status individuals shifted their behavior consistent with these expectations. However, because the present research largely focused on playful contexts, an important test of the expectation-consistent behavior hypothesis will be to extend these results to contexts in which more anti-social behavior is called for. For example, when contexts call for aggression, competitiveness, or out-group derogation, it will be interesting to observe whether elevated status predicts more antisocial tendencies.

Importantly, previous research indicates that teasing and taunting behaviors, when they are elevated to the level of bullying, are related to coercive forms of group influence [44]. The present study assesses teasing and taunting in playful supervised interactions, and as such, status-attainment related to these behaviors is likely the result of appearing consistent with group-based expectations rather than these coercive motives. Still, as much of the work is correlational in nature, our interpretation should be met with caution.

With respect to the correlational nature of the findings, it is not clear if expectation-consistent behavior leads to the attainment of elevated sociometric status, if it maintains elevated status, or if it is simply a confound of other behaviors or psychological states that more directly relate to status. Moreover, we did not assess individual differences in behavior prior to the study, and so we cannot be sure if status-based behavioral tendencies are due to status per se, or some other third variable not measured in the present research. Despite this limitation, it is interesting to speculate about how expectation-consistent behaviors might allow individuals to gain or maintain status by behaving as expected or ideal members of a social group. It would also be interesting to determine when, if ever, violating group norms and expectations is valued in face-to-face social groups.

As well, theoretical accounts of status in face-to-face groups suggest that high-status individuals engage in behaviors that show competence, commitment to the group, or adherence to expectations because these behaviors help individuals gain respect and prestige through their demonstration of value to their social groups. An interesting future extension of the current research involves assessing the extent to which individuals are aware of group expectations when they are in positions of high status. That is, is expectation-consistent behavior a conscious status maintenance strategy? Future research would benefit from considering this possibility.

A related extension of the current findings involves understanding how expectation-consistent behavior may enable the acquisition of elevated social status. Theoretical accounts of status foreshadow this prediction, and suggest that individuals who engage in behaviors that violate group norms are likely to lose prestige and respect from other individuals [3]. Studies of status in organizations corroborate this expectation: Individuals who attain status tend to have personality characteristics suited for their work environments, such as extraversion in socially-oriented organizations [3]. The present studies point to interesting methods for testing this proposition—for example, by experimentally manipulating provocative or affiliative behavior and varying situation-specific expectations.

Of note, these findings should be taken as preliminary because the sample sizes were small when compared with recent changes in standards in the field of social-personality psychology [45]. Thus, all statistical differences reported in the present analyses should undergo future direct and conceptual replications to provide more precise estimates of the potential association between status and expectation-consistent action [46]. Until such time, the findings reported here should be interpreted with extreme caution.

It is also important to consider how expectation-consistent action profiles, among high-status individuals, relate to social dominance. More specifically, are high-status individuals not only more likely to engage in actions consistent with expectations, but also more likely to reject deviant behavior? The aforementioned research indicating that high-status individuals tend to endorse beliefs that justify their position in the social hierarchy [10] is suggestive of this possibility.

Although the present research allowed us to study real behavior in interactions between individuals of varying status in their natural environment, allowing for ecologically valid assessments, status was not manipulated in the present investigation. Future research should uncover whether norm-consistent behaviors emerge when individuals are assigned to a high-status role. Finally, because the participants in the current investigation were young children and adolescents, future research should strive to replicate the present findings with older samples in different social contexts, particularly in organizational relations between people of differing status.

Social interactions in face-to-face groups are a source of potential threat for even the most socially skilled person, as people must negotiate the distribution of shared resources, monitor others' well-being, and regulate their own emotions. Status acts as a heuristic solution in these instances by guiding the actions of high-status individuals toward illustrating competence, group commitment, and willingness to follow prescribed norms, values, and expectations.

## Supporting Information

File S1 **Supporting file contains the following: Study 1 S1.** Raw data for Study 1. **Study 2 S1.** Raw data for Study 2. **Syntax S1.** Syntax for analyses of Study 1 and Study 2.
(ZIP)
